# Emergence of *Klebsiella pneumoniae* Carbapenemase–Producing *K. pneumoniae* with Penicillin-Binding Protein 3 Insertions, Taiwan, 2021

**DOI:** 10.3201/eid3206.260478

**Published:** 2026-06

**Authors:** Tengfei Long, Arianne Lovey, Lillie Sanborn, Yanan Zhao, Zackery P. Bulman, Yi-Tsung Lin, Liang Chen

**Affiliations:** School of Pharmacy and Pharmaceutical Sciences, University at Buffalo, Buffalo, New York, USA (T. Long, A. Lovey, L. Sanborn, Y. Zhao, L. Chen); Retzky College of Pharmacy, University of Illinois Chicago, Chicago, Illinois, USA (Z.P. Bulman); Centre for Infection Control and Division of Infectious Diseases, Department of Medicine, Taipei Veterans General Hospital, Taipei, Taiwan (Y.-T. Lin); Institute of Emergency and Critical Care Medicine, National Yang Ming Chiao Tung University, Taipei (Y.-T. Lin)

**Keywords:** Klebsiella pneumoniae, antimicrobial resistance, bacteria, penicillin-binding protein, aztreonam/avibactam, ceftazidime/avibactam, antimicrobial susceptibility, Taiwan

## Abstract

Carbapenem-resistant Enterobacterales (CRE) are a major global health threat with limited treatment options. Aztreonam/avibactam is a promising therapy against metallo-β-lactamase (MBL)–producing and other CRE, but emerging resistance threatens its effectiveness. Insertions in penicillin-binding protein 3 (PBP3), which are well described in *Escherichia coli*, are linked to reduced aztreonam/avibactam susceptibility but remain poorly characterized in *Klebsiella pneumoniae*. We report clinical *K. pneumoniae* carbapenemase–producing *K. pneumoniae* sequence type 11 isolates carrying a novel PBP3 YRIT insertion, conferring reduced susceptibility to aztreonam/avibactam and ceftazidime/avibactam. Functional and genetic studies suggest that the PBP3 insertion impairs β-lactam binding and, in combination with *bla*_KPC-2_ and other β-lactamases, contributes to reduced susceptibility. Those findings demonstrate the emergence of a PBP3 insertion in a high-risk *K. pneumoniae* clone, underscoring the expansion of this resistance mechanism and the critical need for genomic surveillance and novel therapeutics to identify and treat such infections.

Carbapenem-resistant Enterobacterales (CRE) have emerged as one of the most urgent antimicrobial resistance threats worldwide (*1*). Infections caused by CRE are associated with prolonged hospitalization, limited therapeutic options, and substantial mortality rates. Among CRE, strains producing metallo-β-lactamase (MBL) are of particular concern because MBL enzymes hydrolyze most β-lactams (except for aztreonam), including carbapenems, and are not inhibited by available β-lactamase inhibitors, such as clavulanate, tazobactam, avibactam, relebactam, and vaborbactam. The global spread of MBL-producing Enterobacterales, particularly New Dehli metallo-β-lactamase (NDM)–producing strains, poses a formidable challenge for clinicians and public health systems. Of note, a recent study documented a 461% increase in the age-adjusted incidence of NDM-producing CRE in the United States during 2019–2023 (*2*).

Aztreonam/avibactam is a promising therapeutic option for infections caused by MBL-producing organisms (*3*). Aztreonam is intrinsically stable to hydrolysis by MBLs, and avibactam provides inhibition to coproduced serine β-lactamases, such as extended-spectrum β-lactamase (ESBL) and *Klebsiella pneumoniae* carbapenemase (KPC). The aztreonam/avibactam drug combination overcomes common combined resistance mechanisms in MBL-producers and other CRE, addressing a major therapeutic gap.

Nevertheless, emerging resistance mechanisms threaten the durability of aztreonam/avibactam. One concerning development is the insertion of motifs containing 4 amino acids, most commonly YRIK or YRIN (*4*), into penicillin-binding protein 3 (PBP3). PBP3 is a target of aztreonam and other β-lactam antibiotics, including ceftazidime, cefepime, and cefiderocol (*4*–*7*). Those PBP3 insertions are thought to alter access to the transpeptidase pocket, thereby reducing the activity of PBP3-targeting agents. On their own, PBP3 insertions confer only modest increases in MICs to aztreonam/avibactam and ceftazidime/avibactam (*5*,*6*,*8*,*9*). However, those mutations frequently occur alongside additional β-lactamases (e.g., *bla*_NDM_ and *bla*_CMY_) and other resistance determinants (e.g., *cirA* mutation) (*10*–*12*). Combined, those mechanisms can drive near pan–β-lactam resistance, which can have serious clinical consequences, including treatment failure and patient death (*11*,*12*).

PBP3 insertions predominantly have been described in *Escherichia coli* (*4*); however, the occurrence and clinical significance of PBP3 insertions in *K. pneumoniae*, another multidrug-resistant pathogen and leading cause of healthcare-associated infections, remain poorly understood. A few amino acid substitutions in PBP3 of *K. pneumoniae* have been implicated in increased resistance to aztreonam/avibactam, ceftazidime/avibactam, or ceftibuten/avibactam (*13*–*15*). However, to our knowledge, PBP3 insertions in clinical cases of *K. pneumoniae* have not yet been described in the literature, and the interplay between the PBP3 alterations and β-lactamases warrants further investigation. In this study, we describe 2 clinical cases in Taipei, Taiwan, involving KPC-producing *K. pneumoniae* isolates carrying PBP3 insertions.

## Cases and Methods

### Case 1

The first case occurred in a woman in her mid-70s with a history of heart failure and hypertension who was admitted to a tertiary-care hospital in Taipei for gastrointestinal bleeding in mid-April 2021. She initially received ciprofloxacin for pyuria on the basis of a prior urine culture that had yielded *E. coli*. During that admission, she had acute osteomyelitis of the right great toe diagnosed and underwent sequestrectomy on hospitalization day 14. Cultures from that specimen grew *Enterococcus faecalis* and *Streptococcus constellatus*, and she was treated with moxifloxacin. On hospitalization day 24, a new urinary tract infection developed, and urine culture grew carbapenem-resistant *K. pneumoniae* (isolate no. LC1490). Ceftazidime/avibactam therapy was initiated but was switched to amikacin after susceptibility testing confirmed reduced susceptibility to ceftazidime/avibactam (MIC 8 µg/mL) (Table). After a 7-day course of amikacin, the patient recovered and was discharged. In mid-to-late June 2022, she was readmitted for a right intertrochanteric hip fracture and underwent open reduction and internal fixation. Postoperatively, respiratory failure and shock developed, requiring admission to the intensive care unit. She was treated sequentially with piperacillin/tazobactam and ceftriaxone, with clinical improvement. However, on hospitalization day 18, a sputum culture yielded carbapenem-resistant *K. pneumoniae* (isolate no. LC1491). She received ceftazidime/avibactam for 9 days and recovered.

**Table:** Antimicrobial susceptibility of isolates from a study of emergence of *Klebsiella pneumoniae* carbapenemase-producing *K. pneumoniae* with PBP3 insertions, Taiwan, 2021*

Strain	*bla* plasmids	MIC, µg/mL†								
		IMP	MEM	CAZ	ATM	CAZ/AVI	ATM/AVI	IMP/REL	MEM/VBR	CFDC
LC1489	*bla*_KPC-2_, *bla*_SHV-11_, *bla*_TEM-1_, *bla*_DHA-1_	**128**	**>128**	**>128**	**>128**	8	**8**	0.5	2	0.5
LC1490	*bla*_KPC-2_, *bla*_SHV-11_, *bla*_TEM-1_, *bla*_DHA-1_	**128**	**>128**	**>128**	**>128**	8	**8**	0.25	1	0.5
LC1491	*bla*_SHV-11_, *bla*_KPC-2_	**128**	**>128**	**>128**	**>128**	4	1	0.5	2	0.25
ΔpKPC										
LC1489	*bla*_SHV-11_, *bla*_TEM-1_, *bla*_DHA-1_	**2**	**2**	**>128**	**16**	4	2	0.25	0.25	0.06
LC1490	*bla*_SHV-11_, *bla*_TEM-1_, *bla*_DHA-1_	**2**	**2**	**>128**	**32**	4	4	0.25	0.25	0.06
LC1491	*bla*_SHV-11_	0.25	1	4	2	1	1	0.5	0.25	0.06
LC1491 derivative‡										
LC2124, KPC-21a	*bla*_SHV-11_, *bla*_KPC-21_	**32**	**>128**	**>128**	**>128**	**32**	**>128**	**2**	4	1
LC2125, KPC-21b	*bla*_SHV-11_, *bla*_KPC-21_	**32**	**>128**	**>128**	**>128**	**16**	**>128**	**2**	4	0.5
LC2126, KPC-21c	*bla*_SHV-11_, *bla*_KPC-21_	**32**	**>128**	**>128**	**>128**	**32**	**>128**	**2**	4	1
ΔpKPC-pUC-NDM§										
LC1489	*bla*_NDM-1_, *bla*_SHV-11_, *bla*_TEM-1_, *bla*_DHA-1_	**>128**	**>128**	**>128**	**64**	**>128**	4	**>128**	**>128**	**32**
LC1490	*bla*_NDM-1_, *bla*_SHV-11_, *bla*_TEM-1_, *bla*_DHA-1_	**>128**	**>128**	**>128**	**64**	**>128**	4	**>128**	**>128**	**32**
LC1491	*bla*_NDM-1_, *bla*_SHV-11_	**>128**	**>128**	**>128**	4	**>128**	1	**>128**	**>128**	**32**

*All isolates had PBP3 insertion YRIT, OmpK35 with a premature stop codon at amino acid position 63, and OmpK36 with a glycine–aspartate insertion at amino acid position 134 GD. Bold indicates nonsusceptibility. ΔpKPC, *bla*_KPC-2_ plasmid-cured mutants; ATM, aztreonam; ATM/AVI, aztreonam/avibactam; CAZ, ceftazidime; CAZ/AVI, ceftazidime/avibactam; CFDC, cefiderocol; IMP, imipenem; IMP/REL, imipenem/relebactam; KPC, *Klebsiella pneumoniae* carbapenemase; MEM, meropenem; MEM/VBR, meropenem/vaborbactam; NDM, New Dehli metallo-β-lactamase; PBP3, penicillin-binding protein 3. †Susceptibility determined on the basis of Clinical and Laboratory Standards Institute 2025 breakpoints (*16*), except for ATM/AVI, for which we used EUCAST breakpoint (>4 μg/mL). ‡Selected KPC-21 mutant strains obtained through an in vitro selection experiment. §*bla*_KPC-2_ plasmid-cured mutants carrying a pUC-*bla*_NDM-1_ vector.

### Case 2

The second case occurred in a man in his mid-90s, who was admitted to the same hospital as in case 1 for weakness and turbid urine in late April 2021. His medical history included ischemic stroke with bedbound status and chronic urinary retention managed with a long-term indwelling catheter. Three months before admission, urosepsis caused by multidrug-resistant *E. coli* was diagnosed and treated sequentially with cefepime, meropenem, ceftazidime, and levofloxacin. He was discharged 3 weeks before the late April 2021 admission. Culture of urine collected at admission revealed carbapenem-resistant *K. pneumoniae* (isolate no. LC1489). He was started on ceftazidime/avibactam for urinary tract infection, but susceptibility testing revealed reduced ceftazidime/avibactam susceptibility (MIC 8 µg/mL) (Table). Treatment was then switched to fosfomycin monotherapy for 7 days, and he recovered and was discharged.

### Methods

We performed antimicrobial susceptibility testing of bacterial isolates by broth microdilution, according to Clinical and Laboratory Standards Institute guidelines (*16*). We assessed cefiderocol susceptibility by using iron-depleted Mueller-Hinton media. We performed whole-genome sequencing on a NovaSeq platform (Illumina, https://www.illumina.com) and performed genome assembly, quality control, and identification of multilocus sequence type (ST), capsule (K locus [KL]) and O-antigen locus, resistance genes, and porin mutations, as previously described (*17*,*18*). To assess clonal relatedness among isolates, we used Snippy version 4.6 (https://github.com/tseemann/snippy) to perform core single-nucleotide polymorphism (SNP) analysis, filtering repetitive and recombination regions, as previously described (*17*). As the reference for core SNP analysis, we used the genome of a completely closed ST11 *K. pneumoniae* strain, 2020N17-130, from Taiwan (GenBank accession no. CP129835). That strain is genetically close to isolates from the 2 cases (LC1489–91) but has a wild-type PBP3. 

To evaluate the functional contribution of resistance determinants, we cured the *bla*_KPC-2_ plasmid by using our previously established pCasCure plasmid curing system (*19*), then performed susceptibility testing of cured derivatives. To identify resistance evolution, we conducted multistep in vitro selection experiments under aztreonam/avibactam treatment by using our previously published method (*20*) and performed whole-genome sequencing of resistant mutants to define the underlying genetic changes. We used confocal microscopy of live/dead staining to characterize morphologic changes in *K. pneumoniae* carrying wild-type PBP3 and PBP3 insertions (*21*). In addition, we cloned *bla*_NDM-1_ into a pUC vector and introduced clones into *bla*_KPC-2_–cured strains to assess its effect on antimicrobial susceptibility profiles.

The Institutional Review Board of Taipei Veterans General Hospital provided ethics approval for the clinical data collection of both patients (approval no. 2024-01-004BC); the requirement for informed consent was waived. We deposited raw sequence data into National Center for Biotechnology Information (https://www.ncbi.nlm.nih.gov/bioproject; BioProject no. PRJNA1308160).

## Results

Susceptibility testing showed that the 3 patient strains (LC1489–91) were resistant to imipenem and meropenem and displayed reduced susceptibility to ceftazidime/avibactam (MIC 4–8 µg/mL) and aztreonam/avibactam (MIC 1–8 µg/mL) but remained susceptible to imipenem/relebactam, meropenem/vaborbactam, and cefiderocol (Table). We next conducted conventional PCR and Sanger sequencing to examine whether the reduced ceftazidime/avibactam and aztreonam/avibactam susceptibility was caused by KPC variants (e.g., D179Y) or MBLs. Sequencing results revealed that *bla*_KPC-2_ was the only carbapenemase gene in each of the 3 isolates. The absence of KPC variants or MBLs suggested that other mechanisms caused the reduced susceptibility to ceftazidime/avibactam and aztreonam/avibactam. 

Genomic analysis revealed that all 3 isolates belonged to the high-risk ST11 clone and harbored KL47 and O-antigen locus type 13. Core SNP analysis showed that the isolates differed by an average of 10 (range 8–12) SNPs, indicating a high degree of clonality. All 3 isolates carried the β-lactamase genes *bla*_KPC-2_, *bla*_TEM-1_, and *bla*_SHV-11_. The outer membrane protein OmpK35 contained a premature stop codon at amino acid position 63, and the OmpK36 protein had a glycine-aspartate insertion at position 134. Isolates LC1489 and LC1490 carried an additional plasmidborne AmpC gene, *bla*_DHA-1_ (Table). We suspect that the OmpK defects, combined with different β-lactamases, could partially contribute to the reduced aztreonam/avibactam and ceftazidime/avibactam susceptibility observed in the isolates, which is consistent with findings reported in previous studies (*20*,*22*).

**Figure 1:**
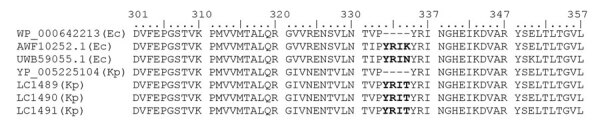
Penicillin-binding protein 3 (PBP3) amino acid alignment of *Klebsiella pneumoniae* carbapenemase–producing *K. pneumoniae* with PBP3 insertions, Taiwan, 2021. Representative *Escherichia coli* genomes with wild-type PBP3 (GenBank accession no. WP_000642213) and YRIK (accession no. AWF10252) and YRIN (accession no. YP_005225104) insertions are shown, as is *K. pneumoniae* genome without insertions. LC1489–91 are patient-derived *K. pneumoniae* genomes with PBP3 YRIT insertions from this study. Ec, *E. coli*; Kp, *K. pneumoniae*.

**Figure 2:**
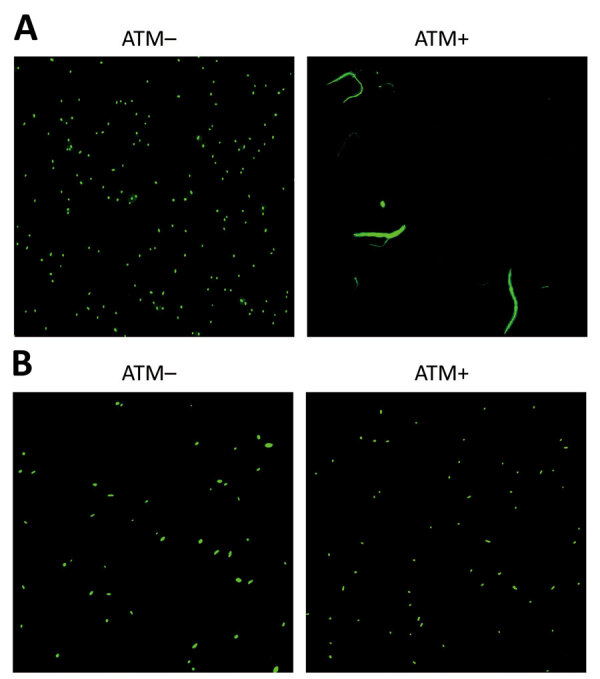
Confocal laser scanning microscopy of *Klebsiella pneumoniae* carbapenemase (KPC)–producing *K. pneumoniae* with penicillin-binding protein 3 (PBP3) insertions with and without ATM treatment, Taiwan, 2021. Isolates on the left (ATM–) were not treated with aztreonam and isolates on the right (ATM+) were treated with 64 µg/mL ATM for 6 hours. A) KPC-producing *K. pneumoniae* with wild-type PBP3 showing elongated and filamentous PBP3 in ATM+, a typical feature of PBP3 inhibition. Isolate used (isolate no. LC6990) was collected from the same hospital as patient isolate in this study (B) and had similar sequence type 11, K locus 47, KPC-2–producing background, and aztreonam MIC (>128 µg/mL). B) KPC-producing *K. pneumoniae* with PBP3 insertion mutations from isolate LC1491 from this study. Note that ATM+ does not show PBP3 inhibition (filamentation) under the conditions of this experiment. Images were acquired using a 63× objective. ATM, aztreonam.

Of note, further mining revealed that all 3 strains contained a 4–amino acid insertion, YRIT, after residue 333 (positions 334–7) in FtsI (PBP3) (Figure 1). Amino acid insertions in PBP3, particularly the tetrapeptides YRIN or YRIK at the same position (aa 334–7), frequently have been reported in *E. coli* and are associated with reduced susceptibility to aztreonam, cefepime, ceftazidime, and cefiderocol, all of which primarily target PBP3. However, a comparable PBP3 YRIX tetrapeptide insertion has not previously been reported in *K. pneumoniae*. We hypothesized that this PBP3 insertion contributed to the reduced aztreonam/avibactam and ceftazidime/avibactam susceptibility observed in those strains. Confocal laser scanning microscopy analysis showed that when treated with aztreonam (≈1/2 MICs for 6 hours), the wild-type PBP3 became elongated and filamentous (Figure 2, panel A), a typical feature of PBP3 inhibition, whereas the PBP3 with insertion mutations remained unchanged (Figure 2, panel B), suggesting that those mutations confer resistance by preventing β-lactam from effectively binding to PBP3.

Of note, on the basis of available data from *E. coli* (*23*), PBP3 tetrapeptide insertions alone are not usually sufficient to confer β-lactam resistance. Those insertions often co-occur with β-lactamases like NDM or CMY, leading to elevated resistance to aztreonam/avibactam and other β-lactams, whereas we detected KPC-2 in our 3 strains. 

To assess the effects of KPC on aztreonam/avibactam susceptibility, we precisely removed the KPC plasmid, then performed susceptibility testing. Curing *bla*_KPC-2_ markedly reduced the imipenem and meropenem MICs, rendering the strains susceptible to carbapenems. However, the *bla*_KPC-2_–cured LC1489 and LC1490 strains remained resistant to aztreonam and ceftazidime because of *bla*_DHA-1_. By contrast, the *bla*_KPC-2_–cured LC1491 strain, which lacked *bla*_DHA-1_, showed an >32-fold reduction in MICs for imipenem, meropenem, aztreonam, and ceftazidime. Curing *bla*_KPC-2_ also resulted in a 2- to 4-fold decrease in the MICs of novel β-lactam/β-lactamase inhibitor combinations, including aztreonam/avibactam, ceftazidime/avibactam, meropenem/vaborbactam, and cefiderocol.

Mutations in the *bla*_KPC_ gene, particularly those located in the Ω-loop, are known to cause resistance to ceftazidime/avibactam, arising through both in vitro selection and in vivo evolution. To determine whether high-level aztreonam/avibactam resistance could be obtained through *bla*_KPC_ mutation, we conducted an in vitro selection experiment to evaluate whether the PBP3 YRIT insertion strain could develop high-level resistance, following a previously published protocol (*20*). We chose strain LC1491 for that experiment because it carried only the *bla*_KPC-2_ carbapenemase gene and the chromosome-bearing *bla*_SHV-11_. After multistep selection for aztreonam/avibactam resistance, the LC1491 strain had a MIC >128 µg/mL (Table). 

To elucidate the underlying resistance mechanism, we isolated 3 resistant colonies from LC1491 (LC2124–26) and performed next-generation sequencing by using the NovaSeq platform (Illumina). Core-genome analysis revealed that the 3 mutants were nearly identical to the parental LC1491 strain, differing by <2 SNPs. Of note, all 3 resistant derivatives harbored a *bla*_KPC-21_ variant, which contained a single-nucleotide substitution (T→A at position 310) resulting in an amino acid change from tryptophan to arginine at Ambler position 105 (Trp105Arg, W105R). That variant corresponds to KPC-21, which was previously identified in a clinical *E. coli* ST131 isolate (*24*) and, in a recent in vitro selection study, was shown to confer resistance to aztreonam/avibactam in *E. coli* when combined with a PBP3 insertion (*25*).

To assess the effects of *bla*_NDM-1_ on the antimicrobial susceptibility profile of PBP3 insertions *K. pneumoniae*, we cloned *bla*_NDM-1_ into a pUC vector (pUC-*bla*_NDM-1_) and introduced it into *bl*a_KPC_-cured LC1489, LC1490, and LC1491. The results showed that acquisition of *bla*_NDM-1_ led to high-level resistance to nearly all tested β-lactams and β-lactam/β-lactamase inhibitor combinations. Aztreonam/avibactam remained the most active agent against the 3 strains, with MICs of 4 µg/mL in pUC-*bla*_NDM-1_–harboring LC1489 and LC1490 (both co-harboring *bla*_DHA-1_) and of 1 µg/mL in pUC-*bla*_NDM-1_–harboring LC1491. In addition, cefiderocol activity was substantially reduced in the *bla*_NDM-1_ constructs, with MICs increasing to 32 µg/mL.

## Conclusions

Taken together, findings from this study identified the emergence of *K. pneumoniae* ST11 strains carrying a novel 4–amino acid YRIT insertion in PBP3, which was associated with reduced susceptibility to both aztreonam/avibactam and ceftazidime/avibactam. Although PBP3 insertions have been more commonly described in *E. coli*, detection in *K. pneumoniae* suggests the possibility for dissemination of this resistance mechanism across species. Of note, *K. pneumoniae* ST11 is a high-risk clone capable of acquiring diverse resistance and virulence plasmids and spreading efficiently (*26*–*28*). Our findings uncover a previously unrecognized mechanism contributing to β-lactam resistance in *K. pneumoniae* and underscore the urgent need for continued genomic surveillance, development of novel therapeutics, and reinforced infection control measures to identify, treat, and prevent such infections.
